# The Outcome of Porcine Foetal Infection with Bungowannah Virus Is Dependent on the Stage of Gestation at Which Infection Occurs. Part 2: Clinical Signs and Gross Pathology

**DOI:** 10.3390/v12080873

**Published:** 2020-08-10

**Authors:** Deborah S. Finlaison, Peter D. Kirkland

**Affiliations:** Virology Laboratory, New South Wales Department of Primary Industries, Elizabeth Macarthur Agricultural Institute, Menangle, NSW 2568, Australia; peter.kirkland@dpi.nsw.gov.au

**Keywords:** Bungowannah virus, foetus, pestivirus, porcine

## Abstract

Bungowannah virus is a novel pestivirus identified from a disease outbreak in a piggery in Australia in June 2003. The aim of this study was to determine whether infection of pregnant pigs with Bungowannah virus induces the clinical signs and gross pathology observed during the initial outbreak and how this correlates with the time of infection. Twenty-four pregnant pigs were infected at one of four stages of gestation (approximately 35, 55, 75 or 90 days). The number of progeny born alive, stillborn or mummified, and signs of disease were recorded. Some surviving piglets were euthanased at weaning and others at ages up to 11 months. All piglets were subjected to a detailed necropsy. The greatest effects were observed following infection at 35 or 90 days of gestation. Infection at 35 days resulted in a significant reduction in the number of pigs born alive and an increased number of mummified foetuses (18%) and preweaning mortalities (70%). Preweaning losses were higher following infection at 90 days of gestation (29%) and were associated with sudden death and cardiorespiratory signs. Stunting occurred in chronically and persistently infected animals. This study reproduced the clinical signs and gross pathology of the porcine myocarditis syndrome and characterised the association between the time of infection and the clinical outcome.

## 1. Introduction

Bungowannah virus is a novel pestivirus identified from an outbreak of a disease in a piggery in New South Wales, Australia, in June 2003 [1]. It is genetically distinct from the other recognised pestiviruses of pigs, namely, classical swine fever virus (CSFV) and atypical porcine pestivirus (APPV) [2,3], with its closest genetic relationship to the recently identified Linda virus [4]. The disease was referred to as the porcine myocarditis syndrome, or PMC, because histological changes in the first affected animals consisted almost exclusively of multifocal, non-suppurative myocarditis, with myonecrosis observed in some cases. The outbreak initially presented as sudden death in 2–3-week-old weaning-age pigs, but soon after the onset, there was a marked increase in the birth of stillborn foetuses and a slight increase in the occurrence of mummified pigs [1]. Cumulative losses in some weeks exceeded 50% of pigs born, and it is estimated that as many as 50,000 pigs died in the initial outbreak. Due to the reproductive effects and disease becoming apparent almost exclusively in the first 2–3 weeks of life, it was presumed to be predominantly the consequence of in utero infection. This hypothesis was supported by the detection of elevated serum immunoglobulin G levels in up to 50% of stillborn pigs, and by the absence of disease in pigs after the immediate postweaning period or in sows farrowing affected litters [1].

The pestiviruses are well-recognised reproductive pathogens with the outcome of infection being dependent on a number of factors, including the stage of gestation that the infection occurs in relation to organogenesis and the development of immune competence [5,6,7,8,9,10,11,12]. CSFV has historically been the main pestivirus recognised to infect pigs (although infections with bovine viral diarrhoea virus (BVDV) and border disease virus (BDV) have been identified), and low virulence strains of CSFV may cause in utero infection and reproductive losses in the absence of disease in the rest of the herd [13,14,15,16,17,18].

Subsequent to the discovery of Bungowannah virus, two more pestiviral infections of pigs have been described: APPV [3] and Linda virus [4]. Both viruses have been associated with congenital tremors in piglets [4,10,19].

While there is strong epidemiological, virological [20] and preliminary experimental [21] evidence that Bungowannah virus is the causative agent of the PMC syndrome, the disease has not been reproduced experimentally. In an accompanying report [22], we described the virological and serological characteristics following the successful infection of the porcine foetus at four different stages of gestation. Regardless of the stage of gestation at which infection occurred, Bungowannah virus was detected in the serum/body fluid and excretions of infected pigs at birth and this was unrelated to the presence or absence of a precolostral Bungowannah virus-specific antibody in these animals. Persistent infections, as described for other pestiviruses, were observed following infection of the dam at 35 days of gestation, as was a chronic infection state where animals that had been presumed to be persistently infected (PI) following infection of the dam at 55 days later seroconverted. Viraemia, virus detection in tissues and viral shedding cleared more rapidly the later in gestation that infection occurred.

The aim of the current study was to determine whether infection of pregnant pigs with Bungowannah virus at different stages of gestation induces the clinical signs and gross pathology of PMC in the progeny that were observed during the initial outbreak. An in-depth description of the histopathological findings will be reported separately.

## 2. Materials and Methods

### 2.1. Study Design

Twenty-four pregnant pigs (22 gilts and two parity-1 sows) with known joining dates were obtained from a piggery known to be free of Bungowannah virus. Full details of the management of these pigs are described in a companion paper [22]. Pregnancy was confirmed using an ultrasound examination prior to selection for the study. The pigs were challenged intranasally at approximately 35 (34–36), 55 (55–58), 75 (72–76) or 90 (90–92) days of gestation (*n* = 6 per group—referred to as D35, D55, D75 and D90; Table 1). These time-points were selected as they were similar to those used in a previous study where foetuses were directly inoculated [21] and they span the gestational age at which the pig foetus is considered to become immunocompetent (70 days). All pregnancies were allowed to proceed to 113 days of gestation; then, all pigs were induced to farrow on day 114 to optimise the collection of blood from piglets prior to suckling.

At birth, all pigs were weighed, their crown–rump lengths recorded, individually identified with ear tags and had their litter identity recorded. They were given an iron supplement intramuscularly between 2 and 4 days of age.

Farrowing outcomes, including the number of progeny born alive, stillborn or mummified, were recorded for each litter. Signs of disease observed throughout the study were documented.

Pigs from infected litters were weaned when they were 21–25 days old. As the presence of Bungowannah virus could still be detected at weaning in several challenge groups, as many pigs as the secure containment facilities could hold were weaned and retained until at least 8 weeks old. Selected pigs from D35 and D55 were kept in the study for a longer period to monitor clinical signs and virological and serological parameters [22]. Throughout the study, pigs that were moribund, not feeding or did not appear to be viable were euthanased.

Pigs from the three litters that did not become infected with Bungowannah virus (on the basis that the virus could not be detected in serum or body fluid for any of the pigs in the litter in a real-time reverse-transcription polymerase chain reaction (qRT-PCR) assay and negative precolostral serology results) were kept as control pigs until 14 to 21 days old, at which time they were euthanased. Because of the logistical considerations associated with the management of breeding sows in a secure containment facility, no mock-inoculated treatment group was included.

Individual pig IDs have been retained in the text where relevant to facilitate comparison between histopathology and virology and serology findings described in related manuscripts [22]. The first number relates to the litter ID and the second relates to the animal ID within the litter (generally in order of birth), e.g., 8-01 indicates the first pig to be born in litter 8.

All piglets were subjected to a detailed necropsy.

The animal studies were approved by the Animal Ethics Committee of the Elizabeth Macarthur Agricultural Institute, AEC Reference No. M09/02 (6 March 2009).

### 2.2. Inoculum

The pregnant pigs were challenged with a total of 5.3–5.8 log_10_ TCID_50_ of Bungowannah virus in 5 mL of phosphate-buffered gelatin saline (2.5 mL per nostril), with the exception of litter 11, where the sow only received approximately 3.8 log_10_ TCID_50_ of the virus. This inoculum was prepared as previously described [22,23].

### 2.3. Statistics

The confounding effect of treatment due to the separate cohorts was considered. The proportions of live, stillborn, mummified (of total born), weaned and lost/died pigs were analysed using a generalised linear model (GLM) as quasi-binomial (allowing for additional variation between sows). Each model included the time of challenge as a fixed effect. For the crown–rump length and birth weight data, a mixed model was fitted, with the time of challenge as a fixed effect, as well as random sow effects.

All calculations were performed with the R statistical software [24] using the glm function to fit the GLM model or the ASReml-R library [25] to fit the mixed models for length and weight.

## 3. Results

All 24 pigs challenged became infected. One pig from D55 failed to farrow and was not pregnant. As previously reported, the foetuses of 20/23 (87%) of the infected dams became infected with Bungowannah virus, as determined by the detection of Bungowannah virus nucleic acid using qRT-PCR in serum or body fluid at birth [22]. Within the 20 infected litters, 226 pigs (including stillborn and mummified foetuses) were born, of which, 225 had been infected in utero [22]. As all infected pigs were viraemic at birth, where individual animals are mentioned, reference is only made to their precolostral antibody level at birth. Three litters (*n* = 42 pigs) were uninfected at birth (as determined by negative qRT-PCR and precolostral serology results) and remained uninfected for the duration they remained in the study (14–21 days).

There were no significant differences in the crown–rump length (*p* = 0.594) and birth weight (*p* = 0.180) of pigs in the four treatment groups and uninfected litters.

### 3.1. Incidence of Stillbirths, Preweaning Deaths and Mummified Pigs

The day of gestation that the pregnant animals were challenged and their reproductive outcomes, including preweaning losses are summarised in Table 1. The greatest effects of infection were observed in the litters of dams infected at 35 or 90 days of gestation. While the use of cohorts confounded results, significant differences were associated with the time of challenge for the proportion born alive (of total) (*p* = 0.029) and proportion mummified (*p* = 0.008), where these values are lower and higher for the D35 group, respectively. For live-born pigs, there were significant differences between challenge groups in the proportion of preweaning losses (*p* = 0.004). The preweaning losses were significantly higher for D35 (there was also a numerically higher mean for D90 compared with D55 and D75; however, this was not statistically significant).

### 3.2. Clinical Signs

Table 2 summarises the reproductive effects, the likelihood of development of persistent infections and the most common clinical signs and necropsy findings observed in the progeny of sows following in utero infection with Bungowannah virus at different stages of gestation.

#### 3.2.1. Group D35

**Figure 1:**
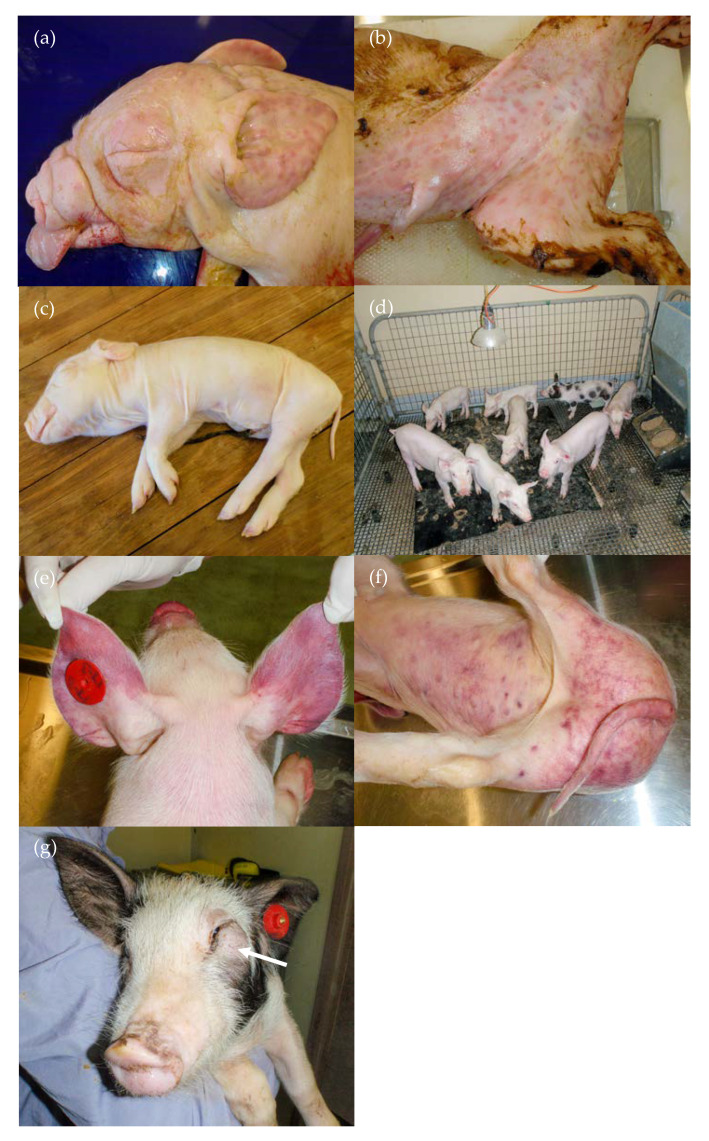
Clinical signs observed in D35 pigs: (**a**) purpura, subcutaneous oedema and absence of facial whiskers (12-09, stillborn); (**b**) purpura (17-11, stillborn); (**c**) subcutaneous oedema—most evident ventrally (12-10, 1 day old); (**d**) remaining pigs at 7 weeks of age with stunting of 6 smaller pigs compared with the 2 larger, non-PI pigs; (**e**) 26-day-old pig with purplish discolouration of the ears and snout (17-03); (**f**) same pig as (e) with purplish discolouration ventrally and on hind-quarters; and (**g**) 16-07 (62 days old) with mass under left eye, resulting in closure of the eyelids (arrow).

The exposure of the pregnant animals at around 35 days of gestation resulted in a high percentage of infected pigs that were seronegative (96%) and presumably persistently infected at birth [22]. Clinically, a high proportion of stillborn (24%) and mummified pigs (18%) and a very high level of preweaning mortalities (70%) resulted (Table 1). The live-born pigs in these litters were often very weak, had difficulties moving from the rear of the sow after birth and some showed a limited ability to suckle. Many died soon after birth or were overlain, presumably as a consequence of their weakness or were euthanased due to their inability to feed. Additional abnormalities observed at birth in both stillborn and live-born pigs included purpura extending over the skin (*n* = 15) (Figure 1a,b) and/or subcutaneous oedema, particularly extending over the head, neck and ventral abdomen (*n* = 9) (Figure 1c). Pigs born with subcutaneous oedema had poor viability. All nine died soon after birth, were weak and were overlain or were euthanased on humane grounds within 3 days of birth. Mild subcutaneous oedema was noted in an additional two pigs at necropsy at 3 and 11 days of age. Of the four pigs with purpura that survived to be weaned, the purpura resolved with age and three of the four appeared to grow normally until weaning, although two developed conjunctivitis at approximately 3 weeks of age. They subsequently died or were euthanased when 26, 34, 35 and 57 days old. Several pigs exhibited neurological signs at birth, including walking into walls (14-04) and screaming, due to an inability to find the udder despite being able to suckle (most of litter 12). One pig (14-04) also developed haemorrhagic diarrhoea in the first week of life that resolved following antibiotic treatment. Three pigs developed diarrhoea in the first week of life, two of which were cross-fostered from their original mother due to milk supply issues, presumably due to poor udder stimulation from weak piglets and were ultimately euthanased (12-02, 12-05) by 2 weeks of age, and a third that had diarrhoea for 3–4 days that resolved following antibiotic treatment and then died suddenly 6 days later (16-02). One 2-week-old pig was euthanased as it failed to grow.

From the five infected litters, eleven pigs survived to weaning and were monitored until approximately 11 weeks old. Of these 11 pigs, nine were considered PI with Bungowannah virus based on ongoing viraemia and shedding of the virus throughout the study until death or euthanasia and negative precolostral serology at birth (14-01, 14-02, 14-04, 14-07, 14-09, 16-07, 17-01, 17-03, 17-04) [22]. The PI pigs grew poorly compared to their two cohorts (17-02, 17-06), which were not PI, based on their precolostral seropositive status at birth [22]. The PI pigs became stunted from soon after weaning (Figure 1d) and exhibited generalised skin pallor from 7 weeks of age. The first pig (17-03) was euthanased within 4 days of weaning after the sudden onset of recumbency; mouth breathing with a small amount of purulent nasal discharge; and purplish skin discolouration of the snout, ears, ventral abdomen and hind quarters (Figure 1e,f). Conjunctivitis was an ongoing problem in two pigs (17-01, 17-04). In addition, three pigs developed firm subcutaneous masses under their eyes between 5 and 9 weeks of age, which forced the lower eyelid dorsally, leaving them unable to open the affected eye (16-07, 17-01, 17-04) (Figure 1g); another developed an erosive lip lesion that resolved following antibiotic treatment at 6 weeks old (14-09). Investigation of the pallor diagnosed anaemia and only the PI pigs were affected. When sampled at 8 weeks old, the packed cell volume of the five affected animals ranged from 8% to 21% (reference interval for weaner pigs 26–41%; [26]). The low levels of circulating reticulocytes (0–0.4%) (corrected reticulocyte percentages <1%) and the normal mean corpuscular volume indicated that the anaemias were non-regenerative and normocytic. In addition, three animals were leukopaenic [26] and three were thrombocytopaenic. While one PI pig survived to 75 days of age (14-01), the remaining PI pigs that did not succumb to other problems were ultimately euthanased due to severe anaemia.

#### 3.2.2. Group D55

There were no clear reproductive effects observed following infection at approximately 55 days gestation (Table 1), with 82% of the infected pigs being seropositive at birth. Two of the stillborn pigs that were seronegative [22] exhibited mild subcutaneous oedema, although the significance is unclear as they were also moderately autolysed. In the immediate postnatal period (first 2 days of life), deaths were attributed to savaging (*n* = 3), overlay (*n* = 3) and runts (*n* = 1). Commencing at about one week after birth, in two litters, four pigs had dark faeces on a rectal swab that was consistent with melaena, suggesting upper gastrointestinal tract bleeding (7-10, 7-11, 10-04, 10-12) and one pig passed bloody liquid at the time of sampling (10-10) (the precolostral antibody titres for 10-04 and 10-10 were 10 and 80 respectively; the three other pigs had suckled prior to the initial sampling). Melaena was detected again on a rectal swab at 14 days of age in one of these pigs (7-11). This pig exhibited pallor, grew poorly compared to its littermates and was euthanased when it failed to gain weight and was unable to compete for food. Another pig (10-12) also grew poorly after melaena was detected at 6 days of age and was euthanased rather than weaned. The third pig (10-04) with melaena grew normally until 13 days old but then grew little in the following week, and at 20 days old, it exhibited pallor and mild jaundice. It was weaned at 28 days, and at this time, it was still growing poorly and exhibiting mild hind limb ataxia. At 19 days old, one pig (7-06; precolostral antibody titre of 1280) was pyrexic (40.7 °C), mildly ataxic with a slight tremor and showed retarded growth compared to its littermates. Despite antibiotic treatment, it was still pyrexic (40.2 °C) 2 days later and continued to exhibit neurological signs, including poor balance with a wide base stance; there was no obvious head tilt or nystagmus. It was noticed that one pig from litter 8 (8-01; seronegative at birth) appeared weaker than the other pigs in its litter in the first couple of days of life and was sometimes slow to move to the teat to feed.

**Figure 2:**
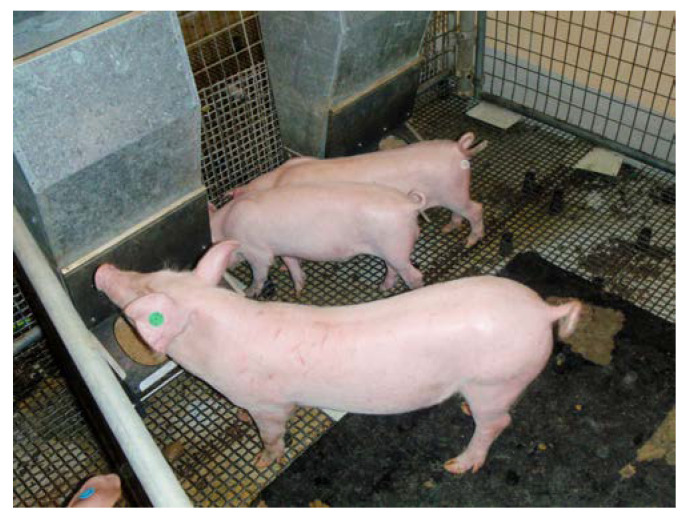
Littermates from litter 8 in D55 at 77 days old—note the stunting of two chronically infected littermates (8-01 and 8-05) compared with the larger non-persistently or chronically infected animal (8-03).

In litters 7 (*n* = 4), 8 (*n* = 3, including 8-01 and 8-05) and 10 (*n* = 5, including 10-01), the pigs with the highest viral loads in serum and oropharyngeal secretions at 2 weeks of age, as well as all pigs surviving to weaning age from litter 11 (*n* = 10), were weaned to clarify whether the ongoing viraemia was due to a persistent infection, given that the dams were infected prior to the expected timing of foetal immunocompetence [22]. All weaned pigs were raised until at least 10 weeks old. Disease was not observed during this period, with the exception of occasional diarrhoea, and at 6.5 and 8 weeks of age, 10-04 had two episodes of pyrexia and lethargy, which responded well to antibiotic treatment on both occasions. The most dramatic clinical difference was the variation in size of the littermates from litter 8 (Figure 2) and litter 10 when 11 weeks old. Across both of these litters, five pigs were live born and seronegative for Bungowannah virus antibody [22]. The two small pigs (Figure 2; 8-01 and 8-05) appeared to be PI on the basis of qRT-PCR results and the absence of antibodies against Bungowannah virus at birth and were markedly stunted compared to their non-PI littermates (seropositive at birth). When 6 to 7 months old, Bungowannah virus RNA could no longer be detected in the serum of these stunted animals; they concurrently developed high antibody titres and their growth rate improved markedly and they are described here as chronically rather than persistently infected [22].

One of the stunted pigs was reared until 6 months of age (10-01), and another two until 11 months of age (8-01 and 8-05). At approximately 3.5 months old, 8-01 and 10-01 experienced an episode of diarrhoea due to a *Salmonella* infection, but otherwise, no disease was observed. At 189 days of age, pig 10-01 weighed 105 kg; at 11 months of age, 8-01 and 8-05 weighed 183 and 153 kg respectively.

#### 3.2.3. Group D75

Losses in the preweaning period were the result of savaging (*n* = 18), overlay (*n* = 2), splay leg (*n* = 1), runt (*n* = 1) and poor mothering/milk production (*n* = 2) and occurred in the first week of life (Table 1). Pigs surviving to weaning did not exhibit any signs of disease and 98% of the infected pigs were seropositive at birth [22].

After weaning, several pigs lost condition compared to others in the group and one developed a skin infection. Otherwise, no disease was observed. No postweaning mortalities occurred and 23 pigs in this group were reared up to 8 weeks of age.

#### 3.2.4. Group D90

Preweaning losses (29%) in this group were higher compared to the D55 and D75 groups (Table 1) and were clinically different from the D35 group. Only 50% of the infected pigs were seropositive at birth, presumably because of the short interval between infection and birth [22]. While some losses due to savaging (*n* = 4) and overlay (*n* = 1) were recorded, some pigs also exhibited tachypnoea and dyspnoea prior to death or euthanasia. Sudden death was recorded for seven apparently healthy pigs between 2 and 5 days of age without any prior signs (19-03, 19-07, 20-04, 22-03, 22-05, 22-06 and 22-08).

Six pigs developed a notable increase in respiratory rate and effort (19-02, 20-06, 21-02, 21-03, 21-09 and 23-11). The earliest onset of respiratory signs was at 4 days and the latest at 7 days old. Three pigs were euthanased due to the severity of their clinical signs. Two pigs, one of which was thinner than its littermates, died 5 days after the detection of an increased respiratory rate. Finally, one pig continued to exhibit an increased respiratory rate when stressed and was thinner than its littermates through to weaning and subsequently to 5 weeks age.

Twenty pigs from this group were weaned and reared up to 5 weeks of age. No disease was evident in these animals.

#### 3.2.5. Uninfected Litters

No disease was observed with the exception of diarrhoea in one pig. The only preweaning losses were due to overlay (*n* = 2), splay leg (*n* = 3), runt (*n* = 1) and accident (*n* = 1) (Table 1). While there was no statistical difference in birth weight between the infected and uninfected litters, the mean weight for the uninfected litters at approximately 7 and 14 days of age was 0.5–0.8 kg and 0.6–1.1 kg greater, respectively.

### 3.3. Necropsy Findings

Table 2 summarises the most common gross pathology findings observed following in utero infection with Bungowannah virus at different stages of gestation. The viral load in tissues at necropsy was dependent on the stage of gestation that infection occurred in, whether the pig was able to mount an immune response in utero and the age of the pig at necropsy [22].

#### 3.3.1. Group D35

Subcutaneous oedema was observed in nine pigs and was most commonly observed ventrally and around the head and neck, giving rise to palpebral oedema and the impression of a Roman nose (Figure 1a,c). Petechiae were observed on the hearts of two stillborn pigs. Purpura (pigs 0 to 3 days old) were most easily identified on the ears, head, ventral abdomen and sometimes on the dorsal body (Figure 1a,b). Petechiae were observed on the pleural surface of the lungs of an overlain pig. White pin-point foci were observed throughout the brain of one stillborn pig (12-09) and a region of presumptive necrosis was observed in the left cerebral hemisphere of another (17-11). Other observations at necropsy in the preweaning period included haemorrhages on the tonsils (*n* = 2) and tongue (*n* = 1), mottled orange liver (*n* = 3) and the absence of facial whiskers (*n* = 1). Red mottled lungs were commonly observed and presumptively attributed to hypostasis. Thoracic and abdominal effusions in stillborn pigs were associated with autolysis. Fibrinous adhesions were observed in the thorax and or abdomen of two pigs that died at 12 and 15 days (both had previously been treated for diarrhoea).

**Figure 3:**
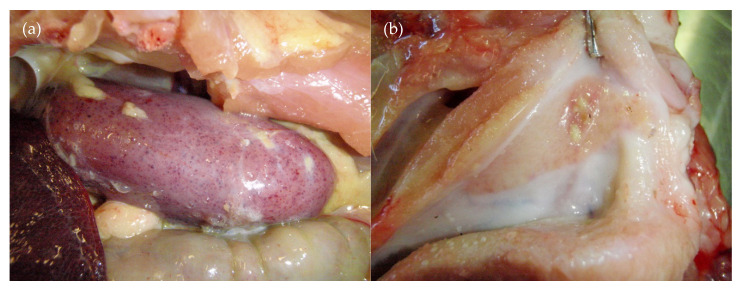
Necropsy findings from D35: (**a**) petechiation of a kidney (17-03—same pig as Figure 1e,f) and (**b**) ulcerated tonsil (16-07).

A range of lesions was observed in the PI pigs that survived past weaning. These animals remained viraemic throughout their life, shed high levels of the virus and had high quantities of the virus in all tissues examined at their necropsies [22]. All became severely stunted, with weights ranging at time of euthanasia from 4.0 kg at 34 days to 13.2 kg at 77 days. Two animals (14-04, 14-09) showed evidence of a bleeding disorder, including the presence of clots in the abdominal cavity and serosanguinous fluid/effusion in the thoracic and/or abdominal cavities; blood streaks were observed in the stomach contents of one of these two pigs. Other changes that indicated a bleeding disorder or vasculitis in the PI pigs included red/haemorrhagic inguinal, mesenteric and/or lumbar lymph nodes in four animals; petechiae were occasionally observed on a number of organs, including the heart (*n* = 3), kidney (*n* = 2) (Figure 3a), liver (*n* = 1), serosal surface of the intestines/colon (*n* = 2), peritoneal surface (*n* = 1), cerebellum (*n* = 1) and oropharynx (*n* = 1). Other abnormalities observed included ulcerated tonsils (16-07) (Figure 3b), increased pericardial fluid (*n* = 4; up to 20 mL), cobblestone pattern to the liver (*n* = 1), mild pulmonary interlobular oedema (*n* = 4) and fibrin tags on intestinal serosa (*n* = 1). The facial mass observed in three pigs (Figure 1g) was firm with an intimate connection between the skin and the subcutis. There was no evidence of wounds in the skin or oral cavity connecting with the mass. Mild subcutaneous oedema was detected in the neck of one pig.

No abnormalities were detected in the two pigs that were not PI (17-02 and 17-06) [22]. At 75 days of age, their mean weight was 30.75 kg. Only low quantities of Bungowannah virus were detectable in these two animals at necropsy [22].

#### 3.3.2. Group D55

Serosanginous pleural and abdominal fluid, as well as mild subcutaneous oedema, was noted in two stillborn pigs that exhibited autolytic changes. Pericardial petechiation was observed in one stillborn pig. Mild interlobular pulmonary oedema and congestion was observed in the lungs of six animals (ages 2–22 days) and was considered to be due to hypostasis. One animal had fibrinous pleural and abdominal adhesions, and another, fibrinous adhesions between the spleen and the liver; both suffered a preweaning death. Other lesions included a cobblestone pattern on the liver, kidney enlargement, oedematous spiral colon and dark blood clots in the colon.

The two chronically infected pigs (8-01 and 8-05) [22] were reared to 11 months of age. Few abnormalities were observed at necropsy. The testes of 8-01 were soft on palpation, fluid collected from the epididymis was of low opacity and sperm were not observed via a microscopic examination, suggesting infertility. The highest viral load detected throughout this study was in the epididymal semen of this animal (9.8 log_10_ copies/mL) [22]. Pig 8-05 was female and penned with 8-01 from 4 months old. From 8 months old, she was observed in oestrus approximately every 3 weeks and did not become pregnant. This was confirmed at necropsy, and an examination of her ovaries showed many follicles and multiple corpora lutea, indicating reproductive maturity.

#### 3.3.3. Group D75

No significant gross pathology was observed for this group. Occasionally petechiae in the heart wall (*n* = 2; stillborn pigs) and consolidated lung lobules (*n* = 6; presumptively due to hypostasis) were observed. Serosanginous pleural and abdominal fluid was noted in two stillborn pigs that exhibited autolytic changes. Bungowannah virus was cleared relatively rapidly from this group, with only low viral loads generally detected after 21–28 days of age [22].

#### 3.3.4. Group D90

Serosanguinous thoracic or abdominal effusions were observed in stillborn pigs with autolytic changes (*n* = 3). Occasional petechiation of the pericardium was also observed (*n* = 1). Two stillborn pigs had fibrin tags in the peritoneal cavity and equivocally enlarged hearts (18-07, 18-08), while another had red tonsils.

**Figure 4:**
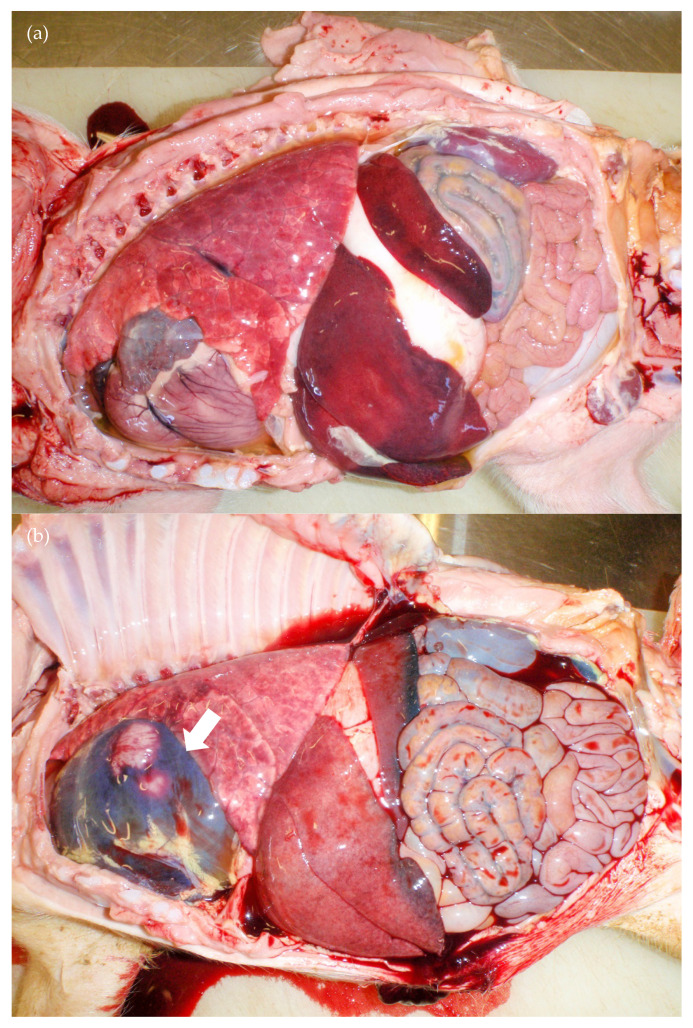
Necropsy findings from D90: (**a**) cardiac and hepatic enlargement (20-06; 7 days old) and (**b**) free blood in the unopened pericardial sac (arrow) and abdomen (19-02; 12 days old).

Of the preweaning mortalities, four of the five pigs observed to have increased respiratory rates exhibited cardiomegaly (20-06, 21-02, 21-03, 21-09) based on subjective observation of an increased base to apex height and increased sternal contact (Figure 4a), and two 5 mm white foci were observed in the superficial myocardium of 20-6. The fifth pig had a marked haemorrhagic pericardial effusion (>50 mL) and blood clots in the abdominal cavity (19-02) (Figure 4b). Thoracic and/or abdominal effusions (*n* = 2), fibrin tags in the thoracic and/or abdominal cavity (*n* = 2), liver enlargement (*n* = 4), reddened inguinal and mesenteric lymph nodes (*n* = 3) and oedema of the spiral colon (*n* = 3) were also observed amongst these five cases. The lungs of the most severely affected pigs did not deflate and a large amount of fluid/froth drained from the lungs after death. Viral RNA was detected in the hearts of all pigs sampled in the first 10 days of life [22]. The pigs that died suddenly (19-03, 19-07, 20-04, 22-03, 22-05, 22-06 and 22-08) appeared to be behaving normally and eating well prior to death. Bruising to suggest they had been overlain was not observed. A range of changes was observed, including subcutaneous oedema of the ventral neck (*n* = 2), pericardial effusion (*n* = 1), fibrin tags in thoracic and/or abdominal cavities (*n* = 2), blood clots in the abdominal cavity (*n* = 2), petechiae and haemorrhages on the kidney (*n* = 1), red/haemorrhagic mesenteric and/or inguinal lymph nodes (*n* = 3), haemorrhages on the spiral colon and caecum (*n* = 2) and oedema of the spiral colon (*n* = 1).

From weaning to termination of the monitoring at 5 weeks of age, few gross lesions were observed. Bungowannah virus was cleared most rapidly from this group with virus shedding and the viral loads in tissues were only at low levels from 11 days of age or often not detected at all [22]. Interlobular pulmonary oedema was observed in two animals and fine fibrin tags were observed in the peritoneal cavity of five animals. The pig that exhibited an increased respiratory rate after exercise and survived to 5 weeks old (23-11) also showed mild interlobular pulmonary oedema and a smaller than expected thymus, but did not have definite cardiac enlargement. However, the ventricular walls appeared thinner than normal, suggesting possible cardiac dilation.

#### 3.3.5. Uninfected Litters

No significant abnormalities were observed at necropsy in pigs from this group. The ages of pigs examined ranged from stillborn to 21 days old. The most frequent changes observed were reddening of the lungs (presumptively due to hypostasis), with some interlobular oedema in four pigs. Other changes observed included petechial haemorrhages on the pericardium, tonsil and skull of an overlain pig, a moderate increase in the amount of thoracic fluid of stillborn pigs, circular skin deficits over the left carpus of two stillborn pigs in litter 9 and moderately enlarged inguinal lymph nodes in a splay-legged pig.

## 4. Discussion

In this study, the clinical signs and gross pathological lesions observed during the outbreak of the porcine myocarditis syndrome were reproduced in the progeny of pregnant pigs experimentally infected with Bungowannah virus. The disease and lesions associated with in utero Bungowannah virus infection at different gestational ages were further characterised. Infection of the foetus at 35 or 90 days gestation resulted in the most severe clinical and pathological effects.

Losses in the field due to PMC were characterised by increased stillbirths, preweaning losses and to a lesser extent, mummified foetuses. At the peak of the outbreak on the farm, on the most severely affected production unit (a gilt unit), preweaning losses approached 50%, stillbirths 40% and mummified foetuses 10–15%, although losses did vary across production units [1,27]. The results of the current study indicated that Bungowannah virus is capable of causing these effects and, depending on the presence of sows at critical stages of pregnancy, could cause significant reproductive losses if introduced into a naïve piggery. The two most critical time-points for exposure of the pregnant pig in relation to disease appears to be around 35 and 90 days of gestation. Our observations following infection at these stages of gestation are consistent with events observed during the early stages of the PMC outbreak, which suggests that the introduction of the virus occurred at a single time-point. The first losses observed were sudden death at 2–3 weeks of age, which is consistent with the outcomes of experimental infection at around 90 days gestation. These initial losses were followed approximately 4 weeks later by a marked increase in the number of stillborn pigs, which would indicate infection at around 40–50 days gestation if 90 days is considered the critical time-point for sudden death at 2–3 weeks of age [20,27].

The description of porcine myocarditis syndrome arose from the fact that early in the field outbreak, affected animals often had an enlarged/dilated heart with evidence of congestive heart failure and non-suppurative myocarditis [1]. Based on the results of this study, it appears that the cardiac enlargement previously reported occurs principally following infection in late gestation (approximately 90 days) and results in a moderate increase in preweaning losses. This was the only challenge group where the previously described [1] multifocal, non-suppurative myocarditis with myonecrosis was observed [28]. Based on these findings, it is clear that the outcome of infection with Bungowannah virus and the associated pathological changes are more extensive than the original field description “porcine myocarditis syndrome” would suggest. Clinically, similar to the field cases, sudden death or increased respiratory rate and effort were observed. Unlike the field situation, cyanosis of the snout and ears and excessive vocalisation before death were not observed during the course of this study. The cardiorespiratory signs and sudden deaths observed have not been reported for CSFV, APPV or Linda viruses, the other pestiviruses primarily infecting pigs. Postnatal deaths are uncommon following infection after 90 days gestation with low virulence strains of CSFV [13,29].

The reproductive effects of in utero infection with Bungowannah virus were most severe following infection around 35 days gestation and are presumably a consequence of the foetus being unable to mount an immune response to the virus at this stage of gestation [22]. These effects included a marked increase in preweaning mortalities (particularly in the first few days of life) and mummified foetuses, as well as a moderate to marked increase in stillbirths, all of which suggests that these effects in the field were the result of infection around 35 days of gestation, reducing by day 55. These clinical findings are similar to those observed following in utero infections with low virulence strains of CSFV that, prior to 70 days gestation, are characterised by high prenatal mortality due to stillborn and mummified foetuses, and increased postnatal mortality with weak-born pigs, although congenital deformities were not observed for Bungowannah virus [13,29,30,31]. The increased levels of stillbirths following exposure of the dam at 55, 75 or 90 days gestation during this study, when compared with industry targets, suggests that Bungowannah virus may have also caused a mild to moderate increase in the stillbirth rate at these time-points, although a greater number of pregnant animals would need to be studied to confirm this finding.

Subcutaneous oedema that spread principally over the head and thorax was observed in a proportion of stillborn pigs during the PMC outbreak [1]. Experimentally, this clinical sign was only observed following Bungowannah virus infection in early gestation (approximately 35–55 days). While purpura was not reported in the field, it was observed in 31% of live and stillborn pigs born to dams infected at around 35 days gestation in the current study. Purpura/petechiae/haemorrhages and subcutaneous oedema have also been described following in utero infection with CSFV in foetuses infected before 70 days gestation [13,29,30,31].

Other clinical similarities between infection with Bungowannah virus at 35 days gestation and in utero CSFV infection prior to 70 days include weak-born pigs that may exhibit or develop poor sucking ability, alopecia, ascites/hydrothorax, petechiae/haemorrhages in organs other than the skin, pallor, diarrhoea, conjunctivitis, anorexia, lethargy or cyanosis of the snout and ears, with some of these changes being observed postweaning [13,29,30,31]. In contrast, the clinical signs following experimental Bungowannah virus infection at 55 days more closely parallel what is observed when in utero infection with CSFV occurs between 70 and 90 days gestation, which is associated with fewer prenatal deaths [29,30,31], although some melaena and diarrhoea was observed in the preweaning period. It is not clear whether the diarrhoea and melaena were increased compared to D75 and D90 pigs due to in utero Bungowannah virus infection or is unrelated.

Following the initial postnatal losses in the D35 group, the next major clinical effect noted following Bungowannah virus infection at either 35 or 55 days of gestation was variability in pig size within a litter postweaning. This disparity in size was noted in those pigs that were born seronegative to Bungowannah virus and, on the basis of PCR results, were considered to be persistently infected [22]. Persistent infections with CSFV are recognised following in utero infection before approximately 70 days gestation and with decreasing frequency up to 90 days gestation [13,29,30]. Clinically, CSFV PI pigs that survive past weaning may present similarly to chronic cases of classical swine fever (CSF), where after an initial, albeit slow recovery, they eventually relapse and die. Initially, they may be indistinguishable from uninfected pigs, but at a variable time (usually weeks to months) after weaning, they often develop severe runting and growth retardation, or “late-onset CSF”, characterised by increasing anorexia and lethargy, pyrexia, conjunctivitis, dermatitis, locomotion disturbances and intermittent diarrhoea [13,15,30,31]. These clinical signs were generally consistent with those observed in pigs infected with Bungowannah virus at D35 that were considered PI. With the exception of a slightly slower growth rate, these pigs often appeared normal while still suckling, but at a variable time after weaning, developed severe growth retardation. Pallor (due to anaemia) and conjunctivitis were the most frequently observed clinical signs. Ataxia and locomotion disturbances were not observed. In contrast, those pigs that were infected at D55 and were born viraemic, seronegative and survived past weaning, while clearly stunted compared to their littermates, were generally disease-free. Whether this would be the case in the field, where there is greater exposure to pathogens, is not clear.

Two of the pigs (8-01 and 8-05) in the D55 group that were initially considered PI were raised until 11 months of age. Unexpectedly, these two animals seroconverted at approximately 6 months of age and cleared their viraemia, at which point, their growth rate increased [22]. After seroconversion, the gilt started cycling at around 8 months of age, which is older than expected and was presumably delayed due to her stunted growth. These animals are similar to the chronically infected pigs described when pigs were experimentally infected in utero with BDV or BDV-like viruses, or BVDV [14,16]. Similar to our study they also noted an improved growth rate after seroconversion. The reason for this improvement is not clear but it is suggested that the effect on growth is the result of a continuing process and not of an irreversible lesion acquired in early development [14].

Stunting is also observed in cattle PI with BVDV, although some animals may appear normal for several years before succumbing to a disease predominantly induced as a result of the persistent BVDV infection. While not observed during the course of this study, it is important to determine whether pigs persistently or “chronically” infected with Bungowannah virus can remain clinically normal for prolonged periods, as these animals are an ongoing source of infection in the herd and their selection as breeding animals could result in disastrous consequences if they were to be introduced into a naïve herd.

Congenital tremors were not observed in this study or in the field following Bungowannah virus infection. Aside from the absence of congenital tremors, the outcomes of infection of Bungowannah virus and CSFV after foetal infection in early gestation are remarkably similar and presumably have a similar pathogenesis. To date, Bungowannah virus has only been identified in Australia, but as its origin remains unknown, it should be considered in the differential diagnosis in reproductive investigations, especially if CFSV is suspected but has been excluded by laboratory testing.

It was not possible to weigh live animals once they reached 5 kg; therefore, all weights postweaning were obtained at the time of necropsy. In retrospect, weekly weights would have been useful to compare animal weights within litters and in relation to infection and serological status at birth. Any comments on stunting in this manuscript are unfortunately subjective rather than objective and relative to that challenge group cohort/littermates only. Additionally, while qRT-PCR results were typically available within a couple of days after sample collection, the serology (peroxidase-linked assay) was batch-tested at a convenient time [22]. The availability of these results may have been useful when selecting animals to follow postweaning, particularly for the D55 group, to better understand the impact of these “chronic” infections on growth rates. As each treatment group was reared individually and at separate times, direct comparisons regarding weight are difficult; in addition, direct comparisons against commercial growth targets are not appropriate.

## 5. Conclusions

This study demonstrates that in utero infection with Bungowannah virus was able to produce the clinical signs and gross pathology observed during the field outbreak and has the potential to be a significant reproductive pathogen of the pig, as well as impacting on postnatal mortality, principally in the first few weeks of life. The outcome of infection was dependent on the stage of gestation at which infection occurs. Maternal infection at around 35 days gestation resulted in high pre- and postnatal mortalities, which reduced when infected at about day 55. Reproductive effects and clinical signs were similar to those observed following in utero infection with low virulence strains of CSFV. Increased preweaning mortalities, cardiorespiratory clinical signs and gross lesions in the heart of the progeny of sows infected at around 90 days gestation suggests that infection in late gestation was required for the development of cardiac pathology. Persistent infections resulted in the development of clinical signs and lesions similar to “late-onset” CSFV.

## Figures and Tables

**Table 1:** Summary of challenge details and reproductive outcomes following the inoculation of pregnant pigs with Bungowannah virus.

Group ^1^	Sow Number (Litter ID)	Day of Gestation at Challenge	Day of Farrowing	Born Alive	Stillborn/AutolySing (%)	Mummified (%)	Total Litter Size	Preweaning Losses (%)	Number Weaned	Comments
D35	4558 (15)	34	112	8	1 (7%)	5 (36%)	14	8 (100%)	0	
D35	4536 (17)	34	114	7	6 (46%)	0 (0%)	13	2 (29%)	5	
D35	4540 (12)	35	111	7	3 (25%)	2 (17%)	12	7 (100%)	0	
D35	4596 (16)	35	114	4	4 (40%)	2 (20%)	10	3 (75%)	1	
D35	4570 (14)	36	114	9	0 (0%)	2 (18%)	11	4 (44%)	5	
**Mean**				**7.0**	**24%**	**18%**	**12**	**70%**	**2.2**	
D55	4261 (10)	55	114	11	1 (8%)	0 (0%)	12	2 (18%)	9	
D55	325 (11)	55	114	13	2 (13%)	0 (0%)	15	3 (23%)	10	Parity 1; pig 11-15 not infected
D55	4409 (8)	56	114	6	2 (25%)	0 (0%)	8	0 (0%)	6	
D55	4268 (7)	58	114	11	0 (0%)	0 (0%)	11	2 (18%)	6	3 piglets savaged
**Mean**				**10.3**	**12%**	**0%**	**11.5**	**15%**	**7.8**	
D75	4294 (3)	73	114	11	0	0	11	3 (27%)	8	
D75	4300 (4)	73	114	7	1	2	10	0 (0%)	7	
D75	4486 (5)	73	114	10	1	0	11	0 (0%)	0 ^2^	10 piglets savaged/poor milk production
D75	4279 (1)	76	110	11	3	0	14	0 (0%)	0 ^2^	11 piglets savaged
D75	4281 (2)	76	114	7	5	0	12	0 (0%)	7	
**Mean**				**9.2**	**16%**	**4%**	**11.6**	**5%**	**7.3**	
D90	739 (18)	90	111	6	3	1	10	1 (17%)	1	4 piglets savaged
D90	735 (22)	90	113	9	2	0	11	5 (56%)	4	
D90	738 (23)	90	114	10	1	0	11	0 (0%)	10	
D90	736 (20)	91	112	9	3	0	12	2 (22%)	7	
D90	2622 (21)	91	114	10	0	0	10	4 (40%)	6	
D90	2623 (19)	92	113	8	0	0	8	3 (38%)	5	
**Mean**				**8.7**	**14**%	**2%**	**10.3**	**29**%	**5.5**	
D35	35 (13)	36	114	13	3	1	17	1 (8%)	12	NI ^3^; Parity 1
D55	4260 (9)	56	114	11	2	2	15	4 (38%)	6	NI; 1 death due to accident
D75	4292 (6)	72	114	10	0	0	10	1 (10%)	9	NI
**Mean**				**11.3**	**10%**	**6%**	**14.0**	**18%**	**9**	

^1^ One sow from the D55 group did not farrow and has been excluded from the table. ^2^ Excluded from mean. ^3^ Litter not infected.

**Table 2:** Summary of the reproductive effects, and most common clinical signs and necropsy findings following in utero infection with Bungowannah virus.

			Clinical Signs		Necropsy Findings	
Day of Gestation at Challenge	Reproductive Effects ^1^	Persistent Infections? ^2^	Preweaning	Postweaning	Preweaning	Postweaning
35 days	Born alive ↓↓↓ Stillbirths ↑↑ Mummified foetuses ↑↑↑ Preweaning deaths ↑↑↑	Yes (>90%)	Purpura, subcutaneous oedema, absence of facial whiskers, weak-born pigs that did not suckle, neurological signs—abnormal behaviour	Stunting, pallor due to anaemia, conjunctivitis, subcutaneous facial skin mass, leukopaenia, thrombocytopaenia, purplish discolouration of the snout and ears, ecchymoses in the skin of the hind-quarter	Purpura, subcutaneous oedema, white foci in the cerebrum, orange/mottled liver, fibrinous adhesions in pleural and abdominal cavities (older animals)	Blood clots and serosanguinous fluid in abdomen/thoracic cavities, petechiae, red/haemorrhagic lymph nodes, ulcerated tonsils, facial mass
55 days	Stillbirths ↑	At birth, 18% were seronegative to Bungowannah virus and apparently persistently infected. The 3 seronegative animals retained postweaning later seroconverted	Melaena, pallor, ataxia	Stunting of apparently persistently infected pigs, occasional diarrhoea	Occasional fibrinous adhesions in thoracic and abdominal cavities	No significant findings
75 days	Stillbirths ↑	No	No disease observed	No disease observed	No significant findings	No significant findings
90 days	Stillbirths ↑ Preweaning deaths ↑↑	No	Sudden death, tachypnoea and dyspnoea	No disease observed	Cardiomegally, liver enlargement, fibrin tags in the thoracic and/or abdominal cavities, blood clots in abdominal cavity, pericardial/thoracic effusions, red/haemorrhagic mesenteric and/or inguinal lymph nodes	No significant findings

^1^ ↑—mild increase, ↑↑—moderate increase, ↑↑↑—marked increase, ↓↓↓—marked decrease. ^2^ [22].
